# The effect of neighborhood deprivation on access to surgical care for pediatric renal tumors

**DOI:** 10.1007/s00383-025-06242-0

**Published:** 2025-11-17

**Authors:** Phillip J. Hsu, Katherine Khosrovaneh, Nick Kunnath, Peter F. Ehrlich, Robin T. Petroze, Samir K. Gadepalli

**Affiliations:** 1 Section of Pediatric Surgery, Department of Surgery, University of Michigan, Ann Arbor, MI USA; 2 Center for Healthcare Outcomes and Policy, University of Michigan, Ann Arbor, MI USA

**Keywords:** Social determinants of health, Outcomes, Childhood kidney cancers, Neighborhood

## Abstract

**Objectives:**

Early identification of pediatric renal tumors improves outcomes. Neighborhood deprivation, a composite measure of social determinants of health, is associated with poor oncological outcomes. We studied whether neighborhood deprivation influenced metastatic presentation or treatment approach.

**Study design:**

Using State Inpatient Databases (SID), we studied children admitted for nephrectomy for a renal tumor in 15 states (2013–2021). Neighborhood deprivation was measured using the Child Opportunity Index 3.0 (COI). Using univariate and multivariate analyses, we examined whether patients presented with metastatic disease and whether they underwent upfront nephrectomy versus nephrectomy after neoadjuvant chemotherapy (delayed nephrectomy).

**Results:**

Out of 1,574 children, 14.2% presented with metastases, and 23.3% underwent delayed nephrectomy. Presentation with metastases and delayed nephrectomy were associated with older age and more complex chronic conditions. Neighborhood deprivation was not significantly associated with presentation with metastases or delayed nephrectomy.

**Conclusions:**

Neighborhood deprivation did not significantly impact the presentation or timing of nephrectomy of pediatric renal tumors; biological factors rather than access to care may be the major drivers of variability. However, administrative databases are limited in their ability to fully inform the study of pediatric renal tumors.

**Supplementary Information:**

The online version contains supplementary material available at 10.1007/s00383-025-06242-0.

## Introduction

Renal tumors identified and treated at lower stages are associated with improved overall and event-free survival [1–3]. It is thus important to understand how access-related factors influence variations in disease presentation and, concordantly, how they are treated. In children with Wilms tumor (WT), the most common pediatric renal tumor, Black race has found to be associated with increased incidence, larger tumor size at presentation, advanced stage disease at presentation, and older age at presentation [4–7]. Hispanic ethnicity has been found to be associated with 37% higher odds of having a distant, rather than localized, SEER stage at diagnosis [4–7]. Although low socioeconomic status has also been associated with slightly delayed diagnosis and lower overall survival from WT, socioeconomic status has not been found to affect disease stage at presentation [8–10].

An additional measure that has been utilized to demonstrate disparities in cancer care in adults is neighborhood deprivation, a measure of the combined impact of several social determinants of health, including socioeconomic status, community relationships, and access to resources and health care [11, 12]. Neighborhood deprivation, which can be measured using composite area-based indices such as the Child Opportunity Index 3.0 (COI), Area Deprivation Index, and Social Vulnerability Index, can be used to obtain a more comprehensive understanding of the availability of care for patients and promote more equitable allocation of resources [13–15]. In a study of the Texas Cancer Registry, neighborhood deprivation was shown to be associated with lower 5-year overall survival in children with WT [16].

Although access-related factors including race and ethnicity impact the stage at which renal tumors present, little is known about the effect of neighborhood deprivation. Thus, measures such as the COI present an opportunity to assess how differences in neighborhood may shape access to the development, diagnosis, and subsequent treatment of pediatric renal tumors. To obtain a greater understanding about whether neighborhood deprivation in the United States influences the presentation of renal tumors, an important predictor of outcome, as well as how they are treated, we analyzed data from children undergoing nephrectomy for malignant renal tumors. Because staging is not directly available in administrative databases for pediatric solid tumors, we instead studied (1) whether tumors presented with distant metastases and (2) whether the patient was treated with an upfront nephrectomy versus neoadjuvant chemotherapy followed by nephrectomy (delayed nephrectomy). Distant metastases represent that the disease is at least Stage IV. In North America, resectable tumors undergo upfront resection, and chemotherapy is only administered after resection. Based on Children’s Oncology Group (COG) recommendations, delayed nephrectomy should represent locally-advanced tumors, those that undergo preoperative neoadjuvant chemotherapy due to challenging surgical characteristics such as large size that is deemed unsafe for resection, involvement of nearby structures or tumor thrombus and intracaval extension (disease that is at least Stage III). This may include tumors with metastatic disease. However, it is possible that adherence to COG guidelines varied, leading to delayed nephrectomy in cases where upfront resection was appropriate. We hypothesized that neighborhood deprivation would increase the likelihood of presentation with metastases as well as the likelihood of treatment with delayed nephrectomy.

## Methods

### Data source

The State Inpatient Database (SID) files are part of a family of databases developed for the Healthcare Cost and Utilization Project [17]. SID includes inpatient discharge records from all hospitals (general adult and pediatric; academic and community) in participating states. The SID is a collection of state-level databases that encompasses all inpatient discharges, regardless of payer, providing a unique view of inpatient care in a state over time, inclusive of all hospitals. As all patients undergoing nephrectomy receive an inpatient admission, we felt that the SID would provide a representative picture of the presence of metastatic disease at the time of admission for definitive nephrectomy or the decision to perform a delayed nephrectomy. As the SID does not include outpatient encounters, we utilized the day of inpatient admission as time point at which metastatic or locally advanced disease was present.

### Participants

Children (age 0–17) undergoing nephrectomy for any malignant renal tumor, including WT and renal cell carcinoma, were identified using State Inpatient Databases (SID). Cases were identified by the International Classification of Diseases, Ninth and 10th Revision, Clinical Modification (ICD-9-CM and ICD-10-CM) codes (Appendix 1) [18]. We used only the admission in which the nephrectomy was performed, as the visit linkage variable was not present for all states, and all states for which the variable was present had a significant proportion of missing visit linkage data. We made the assumption that each admission corresponded to a unique patient, as it would be rare for the same child to undergo two separate admissions and undergo a nephrectomy in each admission. As this was an anonymized database study, there was low potential for bias.

We utilized SID from the following states and years: Arizona, Florida, Iowa, Minnesota, New Jersey, Wisconsin (2013–2021); Colorado, Michigan, North Carolina, Nebraska, New York, Washington (2013–2020); Kentucky (2015–2021); Arkansas (2014–2016); and Maryland (2018–2021). The states were selected as a nationally representative sample. The corresponding years of data were selected as a contemporary cohort with complete data that can be used to define neighborhood impacts.

**Fig. 1:**
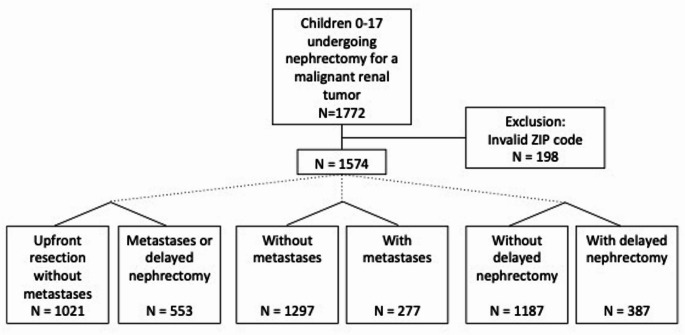
Consort diagram demonstrating the final study population after excluding invalid ZIP codes

We identified 1772 children who underwent nephrectomy for a renal tumor (Fig. 1). 198 children were excluded due to a missing or invalid ZIP code. All other children had complete information and were included.

### Neighborhood deprivation

The selected SID allows for the geographic designation of each patient’s residential ZIP code, allowing cross-walking to COI 3.0 ZIP code data [13]. The COI is a measure of neighborhood opportunity using US census data from 2012 to 2021. In the COI 3.0 ZIP code data, census tract is aggregated to ZIP codes using weights published by the U.S. Department for Housing and Urban Development, where census tracts that are home to a greater proportion of addresses with a given ZIP code receive greater weight. As a composition measure, the COI includes data in the domains of economic opportunities, economic and social resources, health resources, toxic exposures, education, educational and social resources, and healthy environments.

The COI is a relative measure with scores ranging from 1 (lowest opportunity) to 100 (highest opportunity). For each patient, we extracted their 5-digit ZIP code and cross walked to their COI. We then created neighborhood deprivation quintiles based on the COI scores, with the most deprived neighborhoods scored 1–20 and the least deprived neighborhoods scored 80–100.

### Outcomes

Our variables of interest were (1) the presence of metastatic disease on admission for surgery (compared with the absence of metastatic disease on admission for surgery) and (2) treatment with delayed nephrectomy (compared with treatment with upfront nephrectomy without neoadjuvant chemotherapy).

Appendix (1). We aimed to determine whether there are significant differences in the presence of metastatic disease or treatment with delayed nephrectomy between patients living in the most deprived neighborhoods and those living in the least deprived neighborhoods. ICD-9-CM and ICD-10-CM codes used to identify metastases and a history of neoadjuvant chemotherapy are listed in Appendix 1.

### Statistical analysis

The goals of our analysis were to understand the relationship between the presence of metastatic disease and neighborhood deprivation, and the relationship between treatment with delayed nephrectomy and neighborhood deprivation First, we compared the baseline characteristics of patients with and without advanced presentation, including age, sex, race, payer, the presence of a predisposing genetic condition (WAGR, Denys-Drash, Beckwith Wiedemann), the number of complex chronic conditions, American Hospital Association hospital type, year, and COI quintile [19, 20]. We categorized payer as public (Medicare and Medicaid), private (commercial insurance), and other (self-pay, uninsured). We utilized Version 3.0 of the Complex Chronic Conditions ICD code groupings, which are medical conditions expected to last at least 12 months and require specialty pediatric care [19]. Differences were calculated using Chi-square tests. A multivariate risk-adjusted regression model was then used to compare patients with versus without metastatic disease, and patients undergoing delayed versus upfront nephrectomy, using the aforementioned covariates.

## Results

### Demographics

Out of 1574 children, 1021 (64.8%) underwent upfront resection and did not have metastases (Table 1).

**Table 1:** Patient characteristics by presence of metastases and/or delayed nephrectomy

	Total	Upfront resection without metastases	Metastases or delayed nephrectomy	*p*	Metastases	*p*	Delayed nephrectomy	*p*
No. of Patients	*N*=1,574	*N*=1,021	*N*=553		*N*=277		*N*=387	
Age, median (IQR)	3.0 (2.0-6.0)	3.0 (1.0-5.0)	4.0 (2.0-6.0)	0.003	4.0 (3.0-6.0)	<0.001	3.0 (2.0-5.0)	0.140
Sex				0.09		0.26		0.03
Female	823 (52.3%)	518 (50.7%)	305 (55.2%)		151 (54.5%)		221 (57.1%)	
Male	751 (47.7%)	503 (49.3%)	248 (44.8%)		126 (45.5%)		166 (42.9%)	
Race				0.83		0.68		0.66
White	798 (50.7%)	506 (49.6%)	292 (52.8%)		146 (52.7%)		207 (53.5%)	
Black	240 (15.2%)	151 (14.8%)	89 (16.1%)		51 (18.4%)		57 (14.7%)	
Hispanic	251 (15.9%)	161 (15.8%)	90 (16.3%)		38 (13.7%)		67 (17.3%)	
Asian	43 (2.7%)	30 (2.9%)	13 (2.4%)		7 (2.5%)		7 (1.8%)	
Native American	7 (0.4%)	5 (0.5%)	2 (0.4%)		2 (0.7%)		1 (0.3%)	
Other	93 (5.9%)	54 (5.3%)	39 (7.1%)		19 (6.9%)		27 (7.0%)	
Missing	142 (9.0%)	114 (11.2%)	28 (5.1%)		14 (5.1%)		21 (5.4%)	
Payer				0.82		0.97		0.75
Public	706 (44.9%)	453 (44.4%)	253 (45.8%)		121 (43.7%)		179 (46.3%)	
Private	791 (50.3%)	519 (50.8%)	272 (49.2%)		142 (51.3%)		188 (48.6%)	
Other	77 (4.9%)	49 (4.8%)	28 (5.1%)		14 (5.1%)		20 (5.2%)	
Has Predisposing Condition				0.46		0.06		0.12
No	1,540 (97.8%)	1,001 (98.0%)	539 (97.5%)		276 (99.6%)		374 (96.6%)	
Yes	34 (2.2%)	20 (2.0%)	14 (2.5%)		1 (0.4%)		13 (3.4%)	
Complex Chronic Condition Count				<0.001		0.00		<0.001
1	72 (4.6%)	50 (4.9%)	22 (4.0%)		5 (1.8%)		17 (4.4%)	
2	1,195 (75.9%)	806 (78.9%)	389 (70.3%)		201 (72.6%)		263 (68.0%)	
3	233 (14.8%)	127 (12.4%)	106 (19.2%)		53 (19.1%)		80 (20.7%)	
4	52 (3.3%)	27 (2.6%)	25 (4.5%)		13 (4.7%)		19 (4.9%)	
5	16 (1.0%)	8 (0.8%)	8 (1.4%)		3 (1.1%)		6 (1.6%)	
6	6 (0.4%)	3 (0.3%)	3 (0.5%)		2 (0.7%)		2 (0.5%)	
AHA Hospital Type				0.19		0.06		0.42
Adult	804 (51.1%)	503 (49.3%)	301 (54.4%)		161 (58.1%)		206 (53.2%)	
Pediatric	2 (0.1%)	2 (0.2%)	0 (0.0%)		0 (0.0%)		0 (0.0%)	
Both	447 (28.4%)	300 (29.4%)	147 (26.6%)		67 (24.2%)		110 (28.4%)	
Missing	321 (20.4%)	216 (21.2%)	105 (19.0%)		49 (17.7%)		71 (18.3%)	
COI Quintile								
1	268 (17.0%)	172 (16.8%)	96 (17.4%)	0.80	43 (15.5%)	0.60	68 (17.6%)	0.75
2	247 (15.7%)	159 (15.6%)	88 (15.9%)	0.86	49 (17.7%)	0.39	60 (15.5%)	0.97
3	247 (15.7%)	164 (16.1%)	83 (15.0%)	0.58	38 (13.7%)	0.34	58 (15.0%)	0.62
4	340 (21.6%)	220 (21.5%)	120 (21.7%)	0.94	58 (20.9%)	0.83	90 (23.3%)	0.49
5	472 (30.0%)	306 (30.0%)	166 (30.0%)	0.98	89 (32.1%)	0.49	111 (28.7%)	0.64

All P values represent comparison against patients without metastases who underwent upfront resection

Of the 553 children who had metastases and/or underwent delayed nephrectomy, 277 (17.5%) presented with metastases, and 387 (24.6%) received delayed nephrectomy. Children who had metastases and/or underwent delayed nephrectomy were older (median age 4.0 vs. 3.0, *P* = 0.003). Otherwise, all groups were similar regarding sex, race, payer, and the presence of a predisposing genetic condition. Children who had metastases and/or underwent delayed nephrectomy were more likely to have a higher number of complex chronic conditions (*P* < 0.001). Almost all children were treated at a hospital with specialized, comprehensive pediatric care. There was no difference in the type of hospital at which children who had metastases and/or underwent delayed nephrectomy, compared with children who underwent upfront resection and did not have metastases, received surgery. The proportion of admissions did not differ significantly between 2013 and 2021 (Table S1).

### Effect of neighborhood deprivation

Overall, the unadjusted neighborhood deprivation quintile did not differ significantly between children who had metastases and/or underwent delayed nephrectomy, compared with children who underwent upfront resection and did not have metastases (Table 1). After adjusting for age, sex, race, payer, the number of complex chronic conditions, and year, neighborhood deprivation quintile also did not differ significantly between the groups (Tables 2, 3 and 4).

**Table 2:** Risk-adjusted rates of metastases or delayed presentation by COI quintile

Neighborhood deprivation	Rate of metastases or delayed presentation	Odds ratio	*p*
Least deprivation (highest 20% COI)	35.20 (30.73 to 39.66)	Ref	Ref
Below average deprivation	34.89 (29.85 to 39.93)	0.99 (0.73 to 1.33)	0.93
Average deprivation	33.69 (27.82 to 39.55)	0.93 (0.67 to 1.30)	0.69
Above average deprivation	35.66 (29.64 to 41.67)	1.02 (0.73 to 1.43)	0.91
Most deprivation (lowest 20% COI)	36.19 (30.16 to 42.22)	1.04 (0.74 to 1.47)	0.80

**Table 3:** Risk-adjusted rates of metastases by COI Quintile; comparison group = patients without metastases

Neighborhood deprivation	Rate of metastases	Odds ratio	*p*
Least deprivation (highest 20% COI)	18.49 (14.89 to 22.09)	Ref	Ref
Below average deprivation	16.64 (12.73 to 20.56)	0.88 (0.61 to 1.27)	0.49
Average deprivation	15.60 (11.07 to 20.13)	0.81 (0.53 to 1.24)	0.34
Above average deprivation	20.33 (15.23 to 25.44)	1.13 (0.75 to 1.70)	0.57
Most deprivation (lowest 20% COI)	16.56 (11.87 to 21.25)	0.87 (0.56 to 1.35)	0.54

**Table 4:** Risk-adjusted rates of Delaeyd nephrectomy by COI Quintile; comparison group = patients without delayed nephrectomy

Neighborhood deprivation	Rate of delayed nephrectomy	Odds ratio	*p*
Least deprivation (highest 20% COI)	23.64 (19.67 to 27.61)	Ref	Ref
Below average deprivation	26.23 (21.58 to 30.88)	1.15 (0.83 to 1.60)	0.40
Average deprivation	23.50 (18.24 to 28.76)	0.99 (0.68 to 1.44)	0.97
Above average deprivation	24.19 (18.82 to 29.56)	1.03 (0.71 to 1.51)	0.87
Most deprivation (lowest 20% COI)	25.53 (20.05 to 31.00)	1.11 (0.76 to 1.62)	0.60

## Discussion

Although disparities in the stage of presentation of renal tumors in children are known, the potential role of neighborhood deprivation has been understudied. In this study, we found that neighborhood deprivation was associated with no significant difference in the presentation of renal tumors with metastases or the decision to perform a delayed nephrectomy after neoadjuvant chemotherapy. These findings have several implications regarding the care of children with renal tumors.

Prior work evaluating factors that contribute to presentation at an advanced stage has primarily focused on biological factors, differences between countries, and differences in surgical care. Older age, Black race, and Hispanic ethnicity are associated with advanced stage disease [4–7, 21]. Children in the UK with WT have larger tumor size and more advanced tumor stage compared to children in Germany; and children in Eastern Europe had a lower probability of being diagnosed at a metastatic stage compared with those in central Europe [3, 22]. These differences may represent variations in referral patterns by primary care physicians for abdominal masses. In the United States, children with WT who had lymph nodes sampled had improved survival compared to those for whom lymph nodes were not biopsied, potentially representing the effects of variation in surgical practice and experience [23]. However, social determinants of health can also affect presentation of pediatric cancers. Environmental exposures may affect tumor development, and access to healthcare can affect timeliness of presentation. Thus, our study extends existing work to examine how neighborhood deprivation, determined by COI, may be used to identify patients that may benefit from interventions to reduce the effects of location-based disparities.

Of note, the above studies used different databases with heterogeneity in the variables collected, with some (i.e. COG) stratifying disease by stage, risk, and treatment outcomes. While databases such as COG contain detailed cancer data, accessing and analyzing these databases can take significant time and effort. Our study was unique in its use an administrative claims database, which is easier to access and allowed us to characterize neighborhood deprivation. However, use of administrative claims data limited our ability to collect detailed oncologic data, leading to a more exploratory approach of our study. We made the assumption that if patients underwent delayed nephrectomy after neoadjuvant chemotherapy, it may represent locally advanced disease deemed unsafe for upfront surgical resection (disease that is at least Stage III). However, this was a challenging assumption, as it is possible that deviations from COG guidelines occurred, and neoadjuvant chemotherapy was given to patients with Stage I or II disease when upfront resection was appropriate.

Efforts evaluating the association of oncological care and social determinants of health have raised concern that neighborhood deprivation is strongly associated with worse outcomes in both adult and pediatric cancers [4–6, 8, 9, 11]. The stage of solid tumor presentation in children has been associated with social determinants of health in tumors including rhabdomyosarcoma, ovarian tumors, retinoblastoma, and thyroid cancer [24–28]. Specifically, neighborhood deprivation was shown in a study of the Texas Cancer Registry to be associated with advanced stage of presentation in neuroblastoma and sarcoma, but not WT [16]. Our study builds upon this work by showing that, on a national level, administrative claims database does not show that neighborhood deprivation is associated with the presentation of renal tumors with metastases or the decision to perform a delayed nephrectomy after neoadjuvant chemotherapy. This distinction underscores the complexity of factors influencing the presentation of pediatric cancers, as well as the potential limitations of administrative claims databases in studying oncological disease. The mechanisms of how neighborhood disparities affect pediatric cancers are incompletely understood, presenting an opportunity for future research and targeted intervention.

We measured neighborhood deprivation using the COI for several reasons. In a review of literature, we found that it is more widely used in children than other indices, including Area Deprivation Index and Social Vulnerability Index, and was shown to have only minor variation compared to other commonly used indices in children undergoing surgery [29]. Importantly, it also contains economic measures, including economic opportunities and resources; health measures, including health resources and access to healthy food; and environmental exposures, including industrial pollutants and hazardous waste sites; all of which could reasonably contribute to disease stage at presentation.

Our study has several limitations. First, the SID does not contain information about cancer stage, grade, imaging findings, or post-operative pathology. The SID also does not contain information about the specific type of malignant renal tumor (i.e. WT, renal cell carcinoma, or others). This limited our ability to characterize differences in presentation at the most specific level. Although administrative databases are very useful for comparing a wide range of surgical conditions across years and states, they are more limited in evaluating pediatric solid tumors. However, the presence of metastases or locally unresectable disease represents presentation at a more advanced stage, and was associated with known biological factors as expected. Second, children who do not undergo surgery are not included in our database, as we examined only children who received a nephrectomy for a renal tumor. As all malignant renal tumors require surgery as an essential component of definitive treatment, our results are thus only generalizable to children who would benefit from surgical care. Third, our study only captures information at the time of presentation for surgery, is limited by diagnosis and treatment coding, and does not examine outcomes of surgical care, which may be affected by neighborhood deprivation. As other studies have found that social inequality, including neighborhood deprivation, are associated with worse overall survival in WT, further studies are needed to determine whether neighborhood affects outcomes of surgical care [8, 10]. Finally, COI is a measure of community-level disadvantage along several social determinants of health, and it may not provide a full representation of an individual’s social risk or poverty level. However, its inclusion of a broad range of measures across domains of economic status, health resources, and environmental exposures makes it a more robust tool for characterizing how a child is affected by where they live.

Our findings that children with renal tumors do not appear to have disparities in presentation based on neighborhood may suggest that renal tumors could be a case example demonstrating that equitable pediatric cancer may be achievable. Children with renal tumors appear to be treated at specialized centers in a timely manner regardless of neighborhood deprivation, highlighting the need to mitigate disparities in other pediatric cancers. While we hope that our findings support wide availability and standardization of care for children with renal tumors, the data is too limited to comment definitively on lack of disparities. Notably, as our study was unable to account for specific factors including stage and histology, it remains possible that disparities in presentation of renal tumors do exist at a more nuanced level. Additionally, it is important to note that disparities in the care of children with renal tumors may impact measures not explored in our study, including post-operative and long-term outcomes such as recurrence, morbidity, and mortality.

## Conclusions

In our study of children with renal tumors using an administrative claims database, we did not find an association between neighborhood deprivation and presentation with metastases or treatment with delayed nephrectomy. Our findings suggest that, while social determinants of health play an important role in many pediatric cancers, administrative claims data does not show a significant relationship between neighborhood disparity and presentation or treatment received, and suggests that tumor biology may play a more important role. Future analyses with granular data on tumor stage and key post-operative outcomes would offer a clearer understanding of how neighborhood disparities impact the care of pediatric renal tumors.

## Supplementary Information

Below is the link to the electronic supplementary material.

Supplementary Material 1

## Data Availability

No datasets were generated or analysed during the current study.

## Appendix 1

*International Classification of Diseases*,* Ninth and 10th Revision*,* Clinical Modification* (ICD-9-CM and ICD-10-CM) codes used.

Nephrectomy: 0TT00ZZ, 0TT10ZZ, 0TT20ZZ, 0TB00ZZ, 0TB10ZZ, 0TT04ZG, 0TT04ZZ, 0TT14ZG, 0TT14ZZ, 0TT24ZG, 0TT24ZZ, 55.51, 55.4.

Renal tumor: C64, C64.1, C64.2, C64.9, C65, C65.1, C65.2, C65.9, 189.0 189.1 189.2 189.3 189.4 189.5.

Metastases: 197.0, 197.7, C78.00, C78.01, C78.02, C78.2, C78.7.

History of chemotherapy: 3E03305, 3E04305, 9925.

Denys-Drash Syndrome: N04.1, 581.1.

WAGR: Q13.1, 743.45.

Beckwith-Wiedemann Syndrome: Q87.3, 759.89.

Congenital Cardiac Conditions: 745*, 746*, 747*; ICD-10: Q20*, Q21*, Q22*, Q23*, Q24*, Q25*, Q26*, Q27*, Q28*.

*indicates all sub-codes listed under the specified code.

## Abbreviations

COG: Children’s oncology group
COI: Child Opportunity Index 3.0
SID: State inpatient databases
WT: Wilms tumor

## References

[1] Schulpen M, Roy P, Wijnen MHWA, Tytgat GAM, van den Heuvel-Eibrink MM, van Tinteren H, Karim-Kos HE (2022) Incidence and survival of paediatric renal tumours in the Netherlands between 1990 and 2014. Eur J Cancer Oxf Engl 1990 175:282–290. 10.1016/j.ejca.2022.08.021 10.1016/j.ejca.2022.08.021 36174300

[2] Geller JI, Hong AL, Vallance KL, Evageliou N, Aldrink JH, Cost NG, Treece AL, Renfro LA, Mullen EA (2023) Children’s oncology group’s 2023 blueprint for research: renal tumors. Pediatr Blood Cancer 70:e30586. 10.1002/pbc.30586 37477907 10.1002/pbc.30586 PMC10529605

[3] Pritchard-Jones K, Graf N, van Tinteren H, Craft A (2016) Evidence for a delay in diagnosis of wilms’ tumour in the UK compared with germany: implications for primary care for children. Arch Dis Child 101:417–420. 10.1136/archdischild-2015-309212 26948824 10.1136/archdischild-2015-309212 PMC4862069

[4] Lovvorn HNI, Renfro LA, Benedetti DJ, Kotagal M, Phelps HM, Ehrlich PF, Lo AC, Sandberg JK, Treece AL, Gow KW, Glick RD, Davidoff AM, Cost NG, Dix DB, Fernandez CV, Dome JS, Geller JI, Mullen EA (2024) Race and ethnic group enrollment and outcomes for Wilms tumor: analysis of the current era children’s oncology group study. J Am Coll Surg 238 AREN03B2:733. 10.1097/XCS.0000000000000999 10.1097/XCS.0000000000000999 PMC11138877 38251681

[5] Geris JM, Spector LG (2020) Race, ethnicity, and socioeconomic differences in incidence of pediatric embryonal tumors in the united States. Pediatr Blood Cancer 67:e28582. 10.1002/pbc.28582 32672899 10.1002/pbc.28582 PMC7674242

[6] Amirian ES (2013) The role of Hispanic ethnicity in pediatric wilms’ tumor survival. Pediatr Hematol Oncol 30:317–327. 10.3109/08880018.2013.775618 23484868 10.3109/08880018.2013.775618

[7] Apple AN, Neuzil KE, Phelps HM, Li B, Lovvorn Iii HN (2021) Race disparities in genetic alterations within Wilms tumor specimens. J Pediatr Surg 56:1135–1141. 10.1016/j.jpedsurg.2021.02.030 33745745 10.1016/j.jpedsurg.2021.02.030

[8] Chalfant V, Riveros C, Stec AA (2022) Effect of social disparities on 10 year survival in pediatric patients with wilms’ tumor. Cancer Med 12:3452. 10.1002/cam4.5124 35946133 10.1002/cam4.5124 PMC9939224

[9] Collins A, Molina Kuna E, Anderson-Mellies A, Cost C, Green AL (2024) Investigating the impact of tumor biology and social determinants on time to diagnosis and stage at presentation of Wilms tumor. J Pediatr Hematol Oncol 46:147–153. 10.1097/MPH.0000000000002846 38447110 10.1097/MPH.0000000000002846 PMC10956656

[10] Nofi CP, Roberts BK, Brown EG, Rich BS, Kotagal M, Glick RD (2025) Social determinants of health influence on survival in Wilms Tumor, Neuroblastoma, and hepatoblastoma. J Pediatr Surg 60:162216. 10.1016/j.jpedsurg.2025.162216 39947026 10.1016/j.jpedsurg.2025.162216

[11] Bonner SN, Ibrahim AM, Kunnath N, Dimick JB, Nathan H (2023) Neighborhood Deprivation, hospital Quality, and mortality after cancer surgery. Ann Surg 277:73–78. 10.1097/SLA.0000000000005712 36120854 10.1097/SLA.0000000000005712 PMC9974548

[12] Bonner SN, Nuliyalu U, Dualeh SHA, Dimick JB, Nathan H (2023) The combined effect of race, dual-eligibility and neighborhood deprivation on medicare spending after cancer surgery. Am J Surg 226:424–429. 10.1016/j.amjsurg.2023.05.028 37286455 10.1016/j.amjsurg.2023.05.028

[13] Acevedo-Garcia D, McArdle N, Hardy EF, Crisan UI, Romano B, Norris D, Baek M, Reece J (2014) The child opportunity index: improving collaboration between community development and public health. Health Aff Proj Hope 33:1948–1957. 10.1377/hlthaff.2014.0679 10.1377/hlthaff.2014.0679 25367989

[14] Kind AJH, Buckingham WR (2018) Making neighborhood-Disadvantage metrics Accessible - The neighborhood atlas. N Engl J Med 378:2456–2458. 10.1056/NEJMp1802313 29949490 10.1056/NEJMp1802313 PMC6051533

[15] Flanagan BE, Gregory EW, Hallisey EJ, Heitgerd JL, Lewis B (2011) A social vulnerability index for disaster management. J Homel Secur Emerg Manag 8. 10.2202/1547-7355.1792

[16] Cochran ED, Jacobson JC, Nehrubabu M, Qiao J, McCreery S, Chung DH (2024) Social determinants of outcomes disparity among pediatric patients with solid tumor. J Am Coll Surg 238:463–478. 10.1097/XCS.0000000000001010 38258890 10.1097/XCS.0000000000001010

[17] Agency for Healthcare Research and Quality (2022) Health Care Cost and Utilization Project. User Support. https://hcup-us.ahrq.gov/. Accessed 1 Mar 2023

[18] World Health Organization (2016) International Statistical Classification of Diseases and Related Health Problems (9th and 10th ed.); https://icd.who.int/browse10/2016/en. Accessed 1 Dec 2024

[19] Children’s Hospital Association (2024) Complex Chronic Conditions Version 3.0. https://www.childrenshospitals.org/content/analytics/toolkit/complex-chronic-conditions. Accessed 11 Feb 2025

[20] American Hospital Association (2025) Hospitals and Systems. https://www.aha.org/data-insights/hospitals-and-systems. Accessed 11 Feb 2025

[21] Mansfield SA, Lamb MG, Stanek JR, Arnold MA, Ranalli M, Aldrink JH (2020) Renal tumors in children and young adults older than 5 years of age. J Pediatr Hematol Oncol 42:287–291. 10.1097/MPH.0000000000001593 31524665 10.1097/MPH.0000000000001593

[22] Botta L, Didonè F, Lopez-Cortes A, Nieto AC, Desandes E, Hjalgrim LL, Jakab Z, Stiller CA, Zeller B, Gatta G, Pritchard-Jones K, Aitken J, O’Neill L, Youlden D, Hackl M, Ladenstein R, Van Eycken E, Van Damme N, De Camargo B, de Oliveira Santos M, Lima CA, Ramos W, Cabral Formigosa LA, Ferreira dos Santos L, Casale CA, Patrus Pena G, Natívio J, Asturian Laporte C, Santos de Menezes Miranda C, Bastos Daniel C, Nonata de Paulo R, Veneziano DB, Pontes de Aquino A, Fernandes de Souza PC, Valentim Leite R, Valerianova Z, Konstantinov D, Gupta S, Pole JD, Stary J, Sterba J, Hjalgrim LL, Falck Winther J, Paapsi K, Lacour B, Desandes E, Clavel J, Poulalhon C, Ressing M, Truebenbach C, Spix C, Petridou ET, Bouka E, Jakab Z, Garami M, Galasso R, Sampietro G, Sessa M, Piga P, Maule MM, Sacerdote C, Ballotari P, De Santis E, Ragusa R, Torrisi A, Boni L, Rognoni M, Amodio R, Cuccaro F, Bruno D, Russo AG, Gervasi F, Gambino ML, Borciani E, Michiara M, Mangone L, Spagnoli G, Ferretti S, Falcini F, Spata E, Manasse S, Coccia P, Stracci F, Piras D, Pinna P, Bella F, Caldarella A, Intrieri T, Scuderi T, Rizzello RV, Zorzi M, Guzzinati S, Murray D, Matsuda T, Nakata K, Azzopardi MJ, Børge Johannesen T, Dahlen AH, Zeller B, Kowalczyk J, Raciborska A, Ferreira AM, Caldas G, Bucurenci M, Coza D, Zadnik V, Lopez, de Munain A, Almela-Vich F, Jeghalef-El, Karoui N, Marcos-Gragera R, José Sanchez M, Aragonés N, Parra-Blázquez D, Chirlaque MD, Guevara M, Pardo E, Peris-Bonet R, Cañete Nieto A, Carulla M, Lähteenmäki P, Kuehni CE, Redmond SM, Visser O, Karim-Kos H, Stevens S, Irvine L, Stiller C, Gavin A, Fitzpatrick D, Bennet D, Morrison DS, Smith K, Huws DW, Smits S, Gaspar N, Spreafico F, Strauss S, Bailey S, Klein M, Di Cataldo A, Capocaccia R, Polanco A, Greene G (2025) International benchmarking of stage at diagnosis for six childhood solid tumours (the BENCHISTA project): a population-based, retrospective cohort study. Lancet Child Adolesc Health 9:89–99. 10.1016/S2352-4642(24)00302-X 10.1016/S2352-4642(24)00302-X 39855760

[23] Zhuge Y, Cheung MC, Yang R, Koniaris LG, Neville HL, Sola JE (2011) Improved survival with lymph node sampling in Wilms tumor. J Surg Res 167:e199–e203. 10.1016/j.jss.2010.12.026 21324394 10.1016/j.jss.2010.12.026

[24] Roberts B, Cooke-Barber J, Ingram M-C, Danko M, Trudeau M, Glick RD, Short SS, Robertson DJ, Raval MV, Dasgupta R, Rich BS, Committee the AA of PS on SD of SC (2023) Disparities in care of pediatric, adolescent, and young adult patients with solid tumors: A systematic review. Pediatr Blood Cancer 70:e30355. 10.1002/pbc.30355 37066595 10.1002/pbc.30355

[25] Ou JY, Kaddas HK, Alonzo TA, Spector LG, Fallahazad N, Owens E, Collin LJ, Green AL, Kirchhoff AC (2024) Sociodemographic and socioeconomic factors correlate with Late-Stage pediatric hodgkin lymphoma and rhabdomyosarcoma: A report from the children’s oncology group registries. Cancer Epidemiol Biomark Prev Publ Am Assoc Cancer Res Cosponsored Am Soc Prev Oncol 33:1327–1338. 10.1158/1055-9965.EPI-24-0510 10.1158/1055-9965.EPI-24-0510 PMC11446656 39083086

[26] Bownes LV, Stafman LL, Maizlin II, Dellinger M, Gow KW, Goldin AB, Goldfarb M, Langer M, Raval MV, Doski JJ, Nuchtern JG, Vasudevan SA, Beierle EA (2018) Socioeconomic disparities affect survival in malignant ovarian germ cell tumors in AYA population. J Surg Res 222:180–186e3. 10.1016/j.jss.2017.09.013 28988685 10.1016/j.jss.2017.09.013 PMC5742061

[27] Truong B, Green AL, Friedrich P, Ribeiro KB, Rodriguez-Galindo C (2015) Ethnic, Racial, and socioeconomic disparities in retinoblastoma. JAMA Pediatr 169:1096–1104. 10.1001/jamapediatrics.2015.2360 26436436 10.1001/jamapediatrics.2015.2360

[28] Deziel NC, Zhang Y, Wang R, Wiemels JL, Morimoto L, Clark CJ, Metayer C, Ma X (2021) Birth characteristics and risk of pediatric thyroid cancer: A Population-Based Record-Linkage study in California. Thyroid Off J Am Thyroid Assoc 31:596–606. 10.1089/thy.2020.0217 10.1089/thy.2020.0217 PMC8195873 32912083

[29] Stephens CQ, Yap A, Vu L, Saito JM, Barry D, Shui AM, Cockrell H, Cairo S, Wakeman D, Berman L, Greenberg S, Linden AF, Kohler J, Tsao K, Wilson NA (2024) Comparative analysis of indices for social determinants of health in pediatric surgical populations. JAMA Netw Open 7:e2449672. 10.1001/jamanetworkopen.2024.49672 39656457 10.1001/jamanetworkopen.2024.49672 PMC11632545

